# Measuring Cannulation Skills for Hemodialysis: Objective Versus Subjective Assessment

**DOI:** 10.3389/fmed.2021.777186

**Published:** 2021-11-30

**Authors:** Zhanhe Liu, Joe Bible, Lydia Petersen, Prabir Roy-Chaudhury, Judy Geissler, Deborah Brouwer-Maier, Ravikiran Singapogu

**Affiliations:** ^1^Department of Bioengineering, Clemson University, Clemson, SC, United States; ^2^School of Mathematical and Statistical Sciences, Clemson University, Clemson, SC, United States; ^3^UNC Kidney Center, University of North Carolina, Chapel Hill, NC, United States; ^4^(Bill Hefner) VA Medical Center, Salisbury, NC, United States; ^5^Williams S Middleton Memorial Veterans Hospital, Madison, WI, United States; ^6^Transonic Systems, Ithaca, NY, United States

**Keywords:** hemodialysis cannulation, medical simulator, skill assessment, objective metrics, patient care

## Abstract

Lack of cannulation skill during hemodialysis treatments results in poor clinical outcomes due to infiltration and other cannulation-related trauma. Unfortunately, training of patient care technicians and nurses, specifically on the “technical” aspects of cannulation, has traditionally not received much attention. Simulators have been successfully deployed in many medical specialties for assessment and training of clinical skills. However, simulators have not been as widely used in nursing, especially in the context of training clinical personnel in the dialysis unit. We designed a state-of-the-art simulator for quantifying skill for hemodialysis cannulation. In this study, 52 nurses and patient care technicians with varying levels of clinical experience performed 16 cannulations on the simulator with different fistula properties. We formulated a composite metric for objectively measuring overall success of cannulation and compared this metric with subjective assessment by experts. In addition, we examined if years of clinical experience correlated with objective and subjective scores for cannulation skill. Results indicated that, while subjective and objective metrics generally correlated with each other, the objective metric was more precise and better suited for quantifying cannulation skill. Further, the simulator-based objective metric provides several advantages over subjective ratings, including providing fine-grained assessment of skill, consistency in measurement unaffected by subjective biases, and basing assessment on a more complete evaluation of performance. Years of clinical experience, however, demonstrated little correlation with either method of skill assessment. The methods presented for cannulation skill assessment in this study, if widely applied, could result in improved cannulation skill among our PCTs and nurses, which could positively impact patient outcomes in a tangible way.

## 1. Introduction

To receive life-sustaining hemodialysis treatments, patients with end-stage kidney disease (ESKD) need to be cannulated in their vascular accesses at least 3 times a week in order to access their vascular system. Unfortunately, cannulation is a problem ridden procedure for multiple reasons including non-standard geometries of arteriovenous fistulas (AVFs), lack of training opportunities for patient care technicians (PCTs) and a high turnover rate among PCTs in dialysis clinics (1). Lack of cannulation skill results in poor clinical outcomes due to infiltration and other cannulation-related trauma that could potentially lead to an unusable vascular access – a catastrophic event for an ESKD patient. It is estimated that minor infiltration occurs in about 50% of cannulations while major infiltrations occur in 5–7% of cannulations in dialysis clinics (2). Another negative consequence of inadequate cannulation skill is that it increases reliance on Tunneled Dialysis Catheters (TDCs) whether due to not cannulating usable early fistulas (which usually requires greater skill) or due to a temporarily unusable vascular access (3). It has also been reported that injury during cannulation to a maturing AVF is associated with high maturation failure rates (4). Proper cannulation technique can also potentially reduce vessel wall trauma in vascular accesses that could prolong the life of the vascular access (5). In light of these realities, it is imperative that cannulation be performed by skilled clinical personnel in a safe and effective manner since better cannulation skills will positively impact patient outcomes (6). Unfortunately, training of PCTs and nurses, specifically on the “technical” aspects of cannulation, has traditionally not received much attention. Pre-clinical training typically focuses on didactic instruction with “hands-on” training in cannulation comprising only a few attempts on an intravenous (IV) arm mannequin. These “fake arms” are antiquated tools that have limited value for the purpose of teaching cannulation for hemodialysis since they are unrealistic, do not provide quantifiable feedback and cannot simulate a variety of vascular accesses (7, 8). In addition, recent research has brought to light the fact that even though PCTs and nurses may possess several years of experience, they may remain in a state of being “perpetual novices” because of the lack of effective training options (7, 9). The high turnover rate among PCTs further requires that training is both effective and efficient (10).

Simulators have been successfully deployed in many medical specialties for assessment and training of clinical skills (11). One of the key advantages of simulators is their ability to provide objective feedback of task performance. In addition, the trainee has the benefit of practicing skills in an artificial (simulated), safe, low-stakes environment; honing one's skills in this environment has the benefit of instilling confidence in the learner prior to actual clinical practice. Simulators have been demonstrated as being effective for skill assessment and training, particularly in surgical training, with several studies reporting successful transfer of training from the simulator to the operating room (12). Regrettably, however, simulators have not been as widely used in nursing, especially in the context of training clinical personnel in the dialysis unit, due to the fact that current products on the market lack objective assessment and training features and fail to be specifically adjusted for dialysis cannulation applications.

We designed a state-of-the-art simulator for quantifying skill for hemodialysis cannulation (3, 13). In this study, we compare the effectiveness of simulator-based metrics to objectively assess cannulation skill with that of preceptor-based subjective assessment. A highlight of the current work is the formulation of objective metrics to measure the outcome of cannulation, including quality of blood “flashback” and the number of infiltrations. To devise these metrics, we used recommendations from the recent Kidney Disease Outcomes Quality Initiative (KDOQI) guidelines which define good cannulation as one where 2 needles of adequate size are inserted into the AV access at the right depth and angle to facilitate prescribed dialysis and in which this is achieved with minimal pain or no complications (14). The new guidelines also emphasize the need for adapting cannulation skill to “personalized” ESKD care that considers patient preferences in addition to clinical quality measures. To enable superior clinical outcomes in ESKD it is essential that cannulators possess the requisite skills to deal with multiple access types, configurations, and patient life plans.

In this study, 52 nurses and PCTs with varying levels of clinical experience performed cannulation on the simulator. Four types of vascular accesses were included in this study that required different levels of cannulation skill. That is, simulated AV accesses varied in depth, curvature, and diameter. As presented in our previous work (13), the simulator is capable of rendering the key characteristics of hemodialysis cannulation: palpation, needling technique, and flashback dynamics. The aims of this study are: (1) to formulate a comprehensive, objective, and simulator-based metric to assess cannulation skills; (2) to compare the differences between the proposed novel metric with the traditional subjective metric that relies on preceptor-assessed rating sheet; (3) to explore the possible non-linear relationship between cannulation performance and experience levels.

## 2. Methods

### 2.1. Simulator

**Figure 1:**
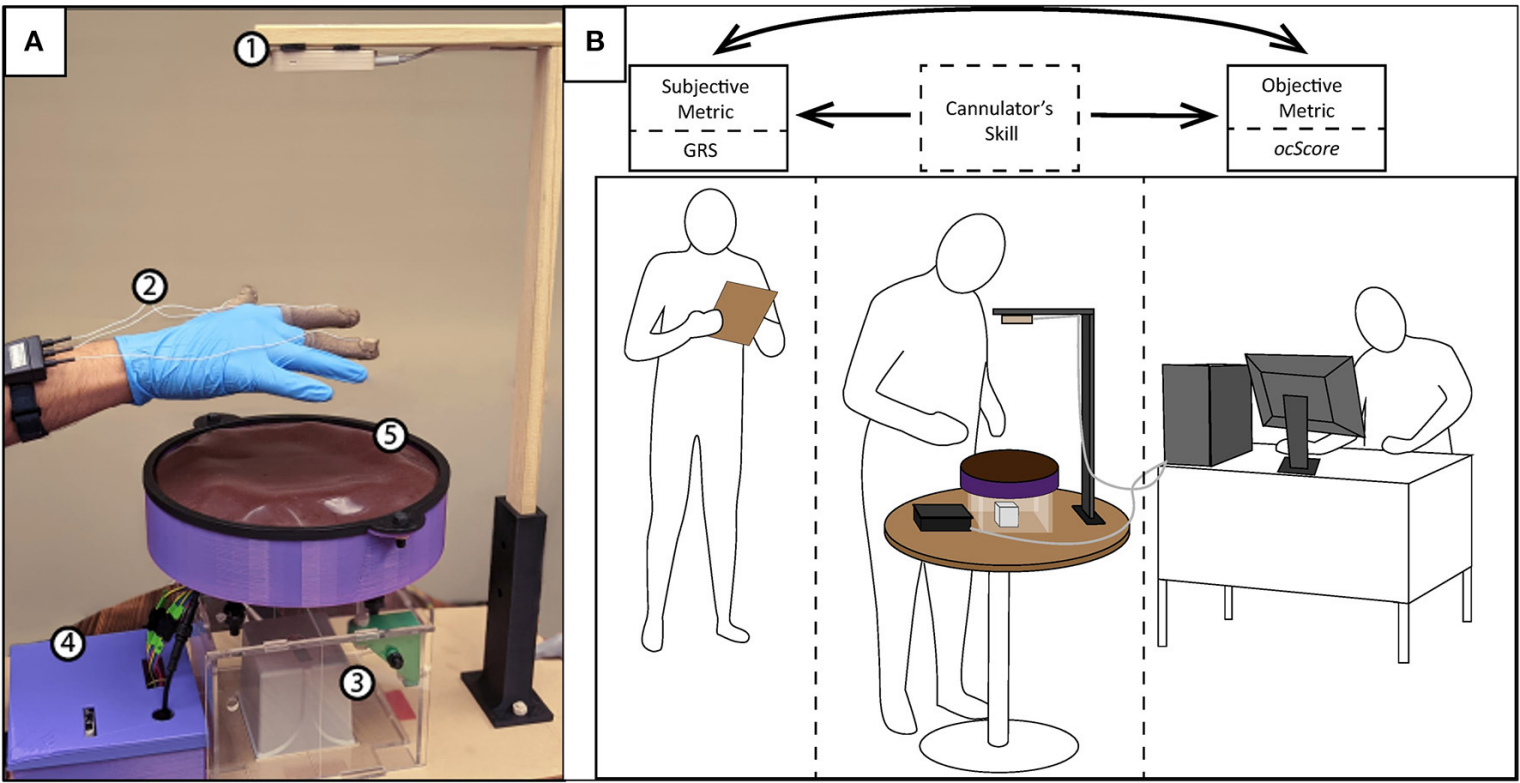
**(A)** The physical simulator with the following components: (1) Leap Motion Controller, (2) FingerTPS, (3) Motion tracker unit, (4) Control Box, and (5) Simulator skin surface; **(B)** Experimental setup with cannulator (PCT or nurse) using the simulator (in the middle), the preceptor using the GRS rating sheet (to the left), and the operator for the simulator (right).

We designed a state-of-the-art simulator with a variety of simulated vascular accesses as well as sensors that measure various facets of cannulation skill (13). Specifically, 4 types of sensors enable multimodal capture of skill: infrared (IR) light sensors, finger force sensing pads, an electromagnetic (EM) motion tracker, and a hand motion recognition camera [details provided in (13); see Figure 1A]. Data from the sensors during human participants experiments were synchronized and integrated via custom-developed software using the *Qt C++* platform.

### 2.2. Experimental Protocol and Participants

**Figure 2:**
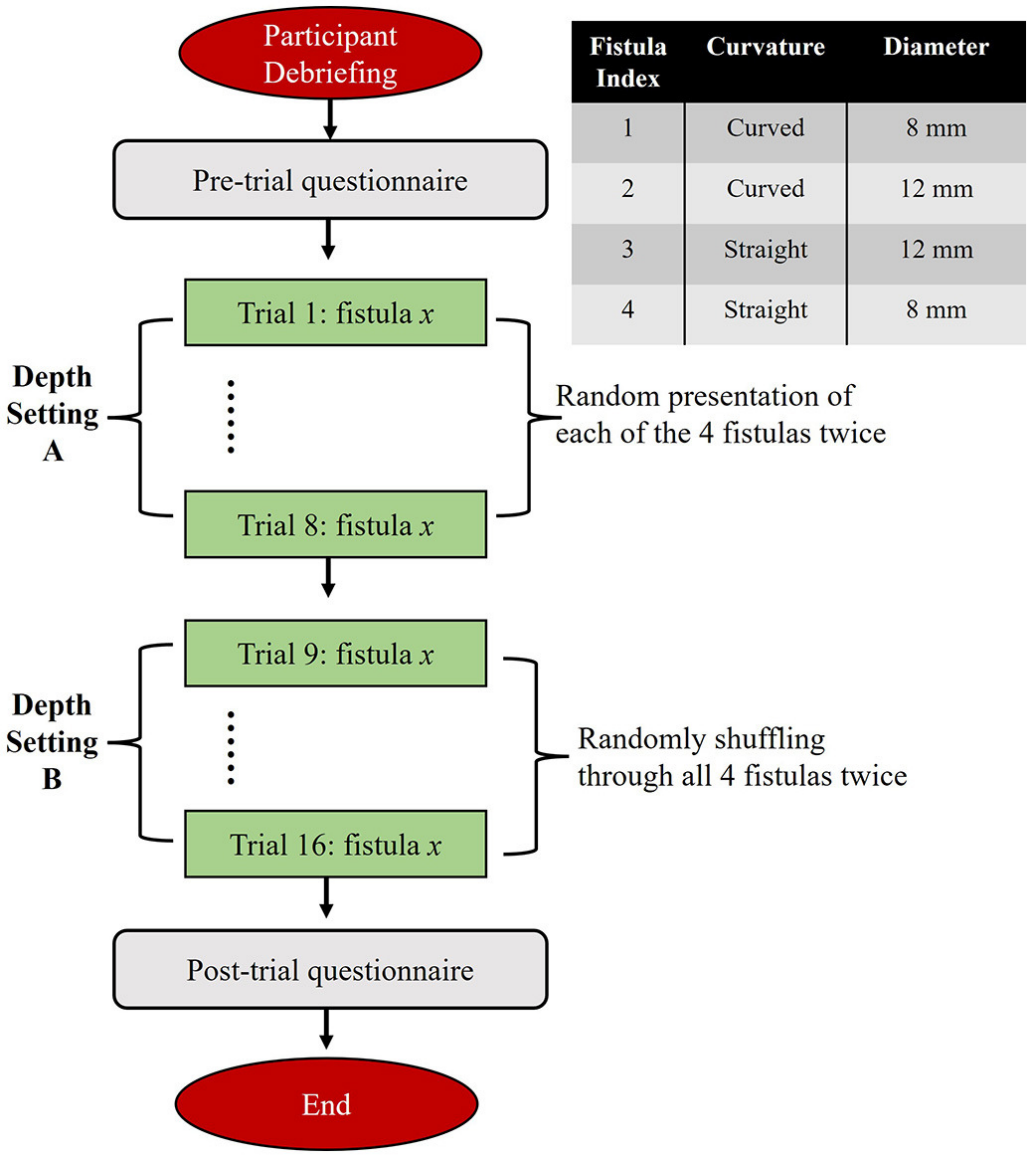
Flow chart of the experimental protocol.

**Figure 3:**
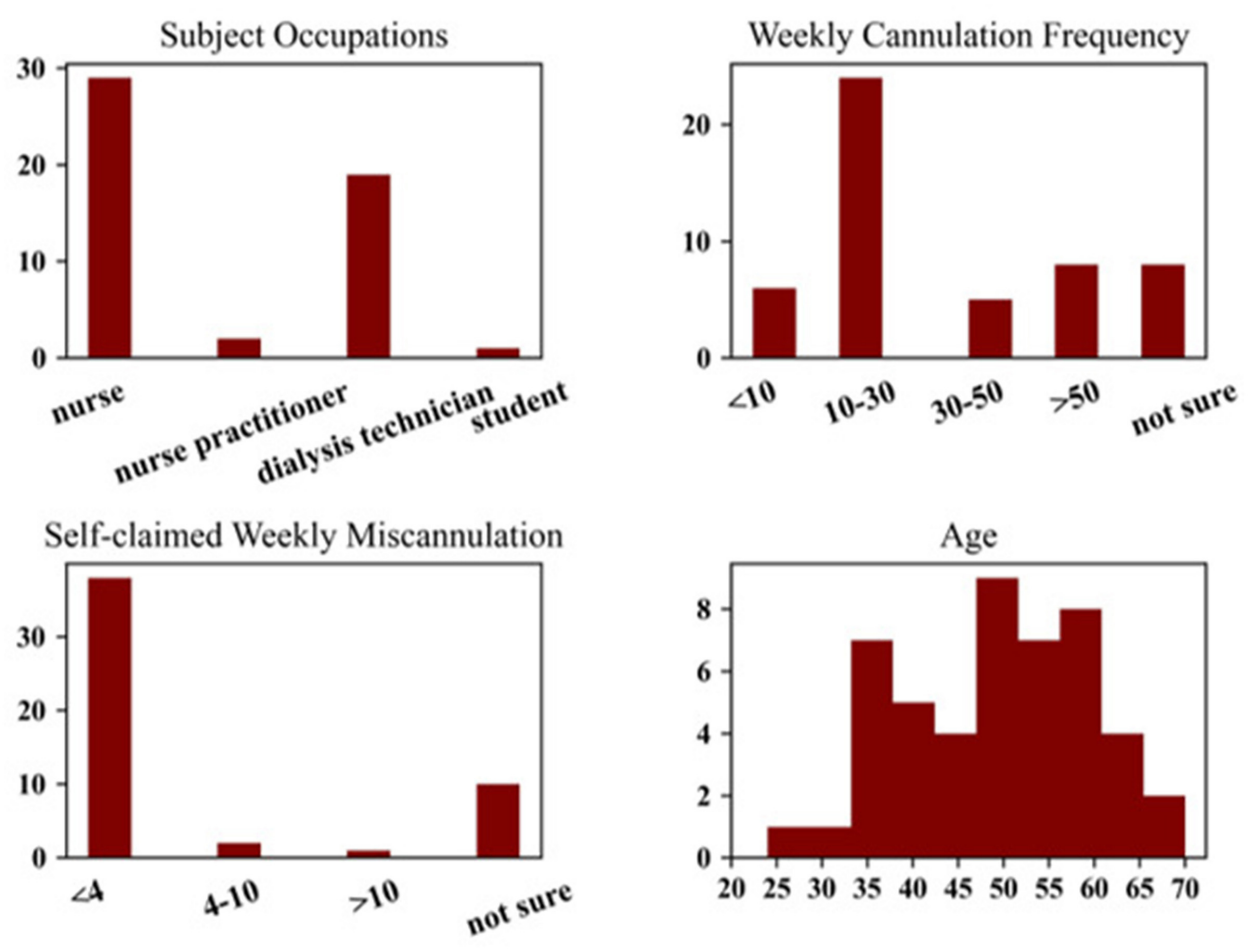
Demographic information of the study population.

**Figure 4:**
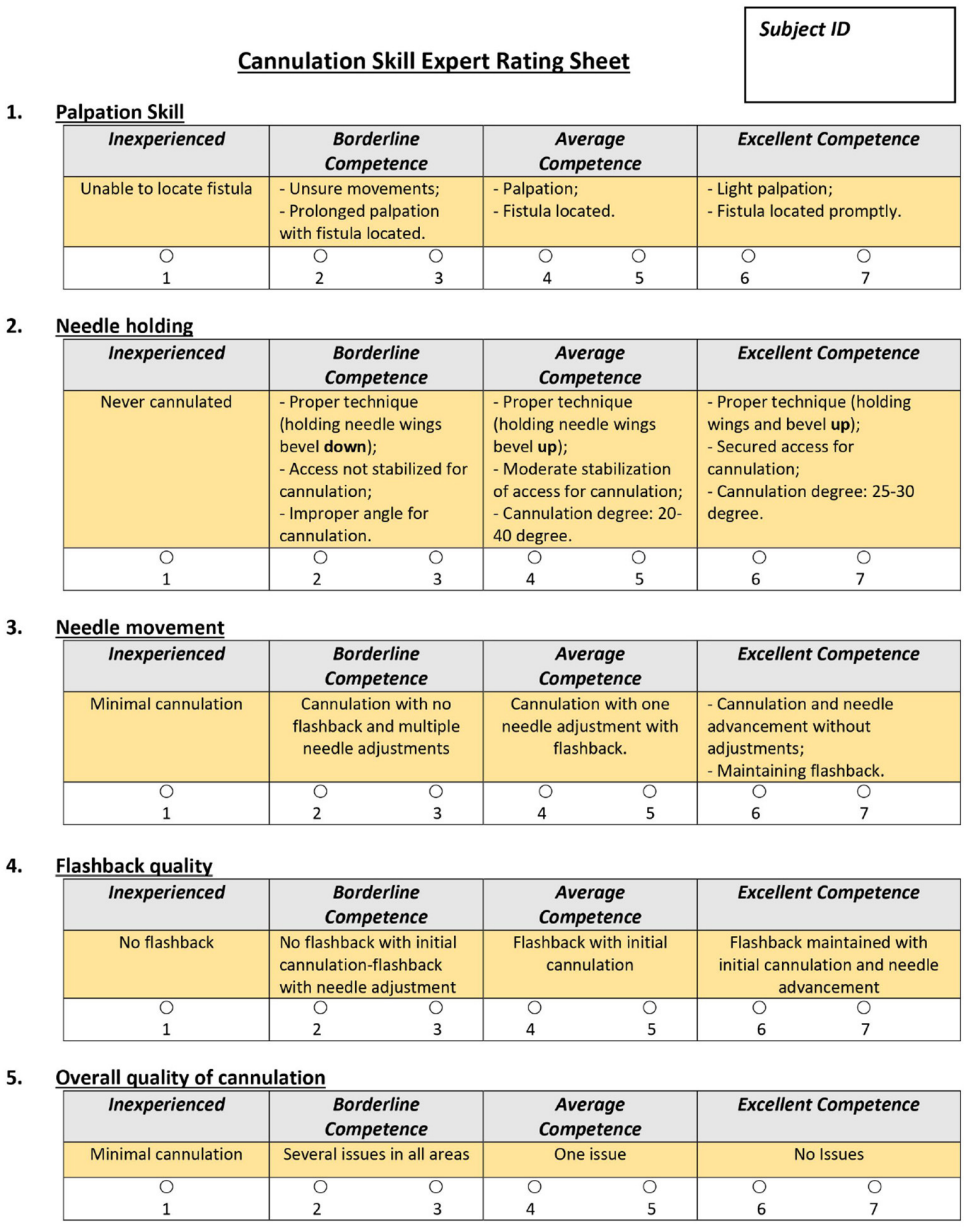
GRS Rating Sheet.

In this study, 52 healthcare professionals with some degree of clinical experience in cannulation, were recruited at a regional ESKD meeting. The experimental setup is depicted in Figure 1B with a corresponding flow diagram of the protocol in Figure 2. Upon arrival and after providing informed consent, participants completed a brief survey regarding their background and the nature of their clinical experience pertinent to hemodialysis cannulation. A summary of participant demographics is presented in Figure 3. In the experiment, each participant was asked to cannulate an artificial access on the simulator. The simulator contained 4 different fistulas with various geometrical and physical characteristics (e.g., diameter, curvature, strength of palpable “thrill”). Each fistula was presented (cannulated) 4 times in random order for a total of 16 trials. Further, during the first 8 trials, one skin thickness was used to simulate either superficial (3 mm) or deep access (4 mm). The latter 8 trials were conducted using the other simulated skin thickness. After all 16 trials were completed, an expert preceptor who observed all 16 cannulations rated the participant's performance on a global rating sheet (GRS) (see Figure 4). The dataset comprised a total of 816 participant cannulation trials; trials from subject A1 were excluded for testing purposes and another 3 trials were excluded due to unavailability of sensor data. Consequently, a total 813 trials were analyzed and presented in this study. Ethics approval for this study was granted by the Greenville Health System (Greenville, SC) Institutional Review Board.

### 2.3. Objective Metrics

To create objective metrics that quantify the outcome of cannulation as defined by the KDOQI guidelines, we define 4 metrics that will be integrated into one composite outcome metric. All metrics are based on the sensor system described in (13) that measures needle location within the simulated fistula, including potential infiltrations. As defined previously (13), *D*_*flash*_ measures the sum of the durations of all flashback periods, including cases where multiple flashbacks were obtained; *T*_*entry*_ is the timestamp when the needle punctures the simulator's skin; *T*_*end*_ is the timestamp of the end of each trial. These values were identified through needle position data, IR signal feedback, and the video recording [details of signal processing can be found in (13)].

The first metric, *flash efficiency* (*eff*), is designed to measure the efficiency with which participants obtained flashback during the whole task. The definition of *flash efficiency* is:


$$eff=\frac{D_{flash}}{T_{end}-T_{entry}}$$

The second metric, *number of attempts* (*#att*), counts the number of times the needle was pulled out and reinserted into the simulator after the first insertion. By default, the metric is instantiated at 1 since every trial has at least one attempt. A number greater than one–more than one insertion attempt–is undesirable per KDOQI guidelines.

The third metric, *stb*, is a binary indicator regarding attainment of stable flashback: 0 stands for failure to maintain stable flashback and 1 stands for the ability to maintain stable flashback. The criteria of stable flashback is that there is at least 2 s of flashback without any interruption until the end of a trial (i.e., when participants signal completion of trial to operators).

The last metric, *number of infiltrations* (*#infil*), estimates the number of times the needle perforated the vascular access by detecting the number of times flashback occurs and then disappears during the insertion process. Each occurrence of this behavior is counted as one infiltration. Per KDOQI guidelines, infiltration ought to be avoided because it often results in bruising and/or pain in addition to adverse clinical complications (14). Note that it is entirely possible for a subject to record multiple infiltrations but to ultimately obtain stable flashback.

Based on these metrics that measure specific aspects of cannulation outcome, we formulated a composite metric for measuring overall success of cannulation. As such, *ocScore* is defined as:


ocScore=eff(1-0.25(I[#att>1]+I[#infil>1]+I[~stb]))

The range of *ocScore* is [0,1]. As per the KDOQI guidelines, perfect cannulation may be defined as one insertion attempt with stable flashback and no needle infiltrations while minimizing patient pain. Ideal cannulation may be expected when flash efficiency is at 100%, with only one insertion attempt, stable flashback, and no infiltration. However, due to the definition of flash efficiency, it is impossible to reach 100% efficiency. Effective cannulation, however, will yield *ocScore* values closer to 1. Note also that adverse events like infiltration and/or more than one insertion attempts are penalized in how *ocScore* is formulated, as these are errors that should be avoided. From a patient perspective, the quantities measured toward computing *ocScore* have implications for patient pain and comfort. That is, one or more adverse behaviors (e.g., more than one cannulation attempt) results in real pain and discomfort for the patient. One of the primary reasons for formulating this composite metric is precision assessment of cannulation outcome on a continuum. Such a metric will be valuable for large-scale assessment of skill relative to a population of cannulators.

### 2.4. Subjective Metrics

In addition to objective assessment of cannulation performance on the simulator, we also employed subjective assessment by expert nurses during cannulation on the simulator. Four expert nurse educators served as preceptors in our study to assess cannulation skills. (Each participant was assessed by one preceptor). After 16 trials were completed, a nurse educator gave a global rating sheet (GRS) score for each subject in the 5 categories, including an overall measure of skill (see Figure 4).

## 3. Results

In this section, we present results from the study in the following order: first, we detail objective outcome metric results; next, we present the relationship between subjective (i.e., GRS) and objective (i.e., *ocScore*) metrics; finally, we demonstrate the relationship between participants' clinical experience and their skill metrics.

### 3.1. Objective Outcome Metrics

**Figure 5:**
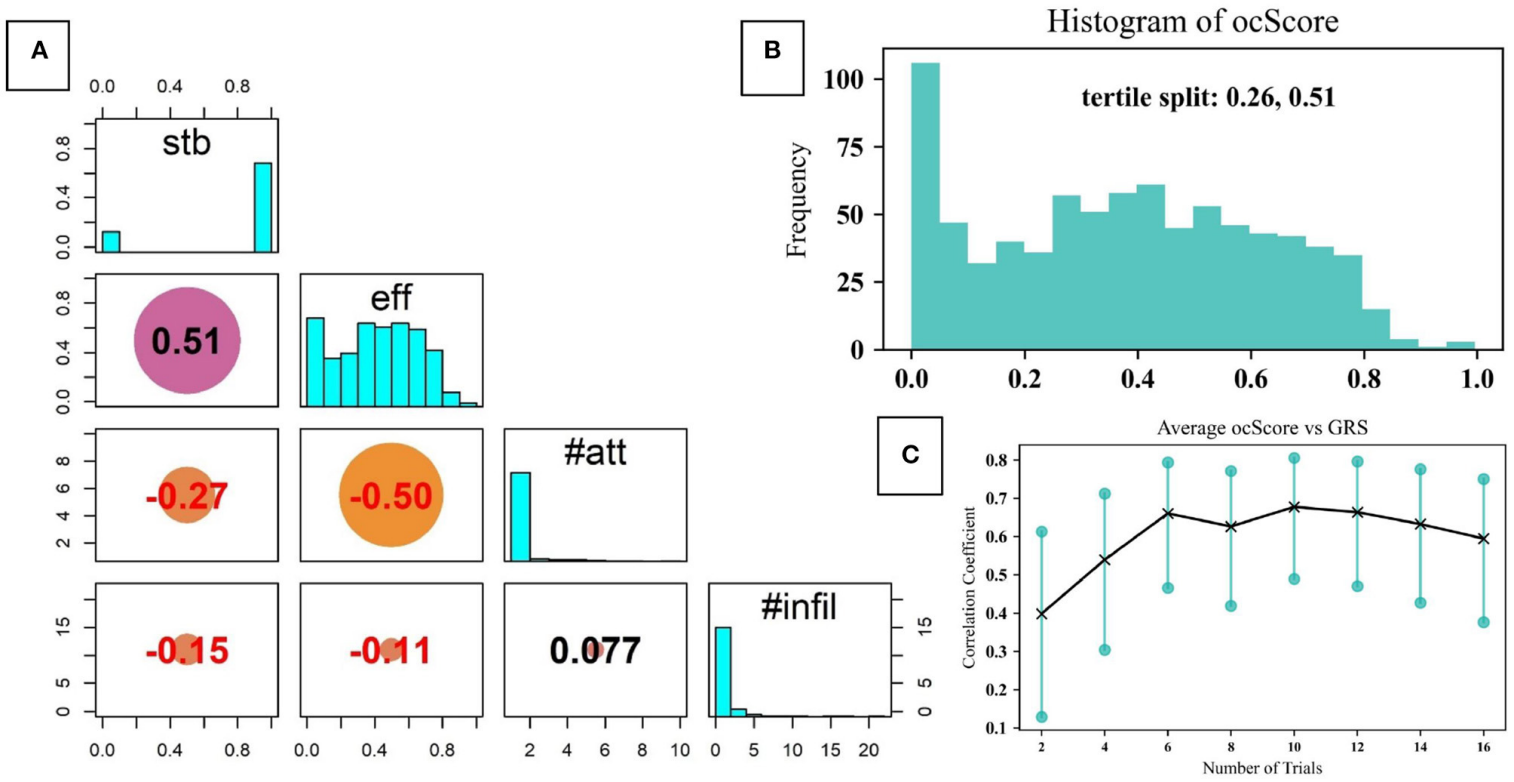
**(A)** The distribution of outcome metrics and their correlation coefficients with each of the other metrics; **(B)** Distribution of ocScore values for all the dataset, divided into tertiles; **(C)** Correlation between ocScore and GRS as a function of number of trials.

Figure 5A depicts the individual distributions of each of the outcome metrics (*eff*, *#att*, *stb*, and *#infil*) as well as the correlations between these metrics. The diagonal panel displays the distribution of each outcome metric, while their correlations with other metrics can be determined by the non- diagonal elements.

As can be noted, in 81.8% of the trials stable flashback was achieved. This reveals that in approximately 82% of the trials, not only did participants cannulate on the correct fistula (since only one was “on” at a time), but that they were successful in receiving stable flashback. Note, however, that this metric measures only if stable flashback was ultimately reached; it does not independently account for the errors and inefficiencies before flashback was ultimately achieved. To get a better picture of this, we turn to the other outcome metrics. As mentioned previously, *eff* measures the efficiency with which participants obtained blood flashback. Only 40.0% of the trials scored over 0.5 in flash efficiency, indicating that the majority of trials were only moderately efficient. That is, the time and needle motion expended to obtain flashback was not optimal.

Furthermore, 15.9% of the trials had more than 1 attempt. These were the times when the participant clearly pulled out the needle from the skin and reinserted. This is undesirable since ideal cannulation should only involve one insertion attempt. A critical finding was that 54% of the trials had at least one infiltration while 24.1% of the trials recorded more than 1 infiltration.

Figure 5A also demonstrates the correlation coefficients between individual outcome metrics. As can be expected, there is a relatively high correlation between stable flashback and flash efficiency (0.51). Similarly, there is a negative correlation between the number of attempts and flash efficiency (–0.5).

The composite objective outcome metric, *ocScore*, was formulated to facilitate precise measurement of cannulation performance based on outcomes. Figure 5B presents the distribution of *ocScore*. Based on this, the tertile splits are at 0.26 and 0.51. As such, performance can be ordered as low skill, moderate skill and high skill from the first to last tertile, respectively. It can also be noted that 38 trials had a rating of zero in flash efficiency, which corresponds to the failure of obtaining any flashback during the trial. This can be caused by misjudging which fistula was on, poor needling technique (inserting at an improper angle and location) and/or needle motion. This metric allows for the relative placement of a cannulation trial along the distribution of *ocScore*, enabling precise assessment of skill.

### 3.2. Relationship Between Objective and Subjective Metrics

**Figure 6:**
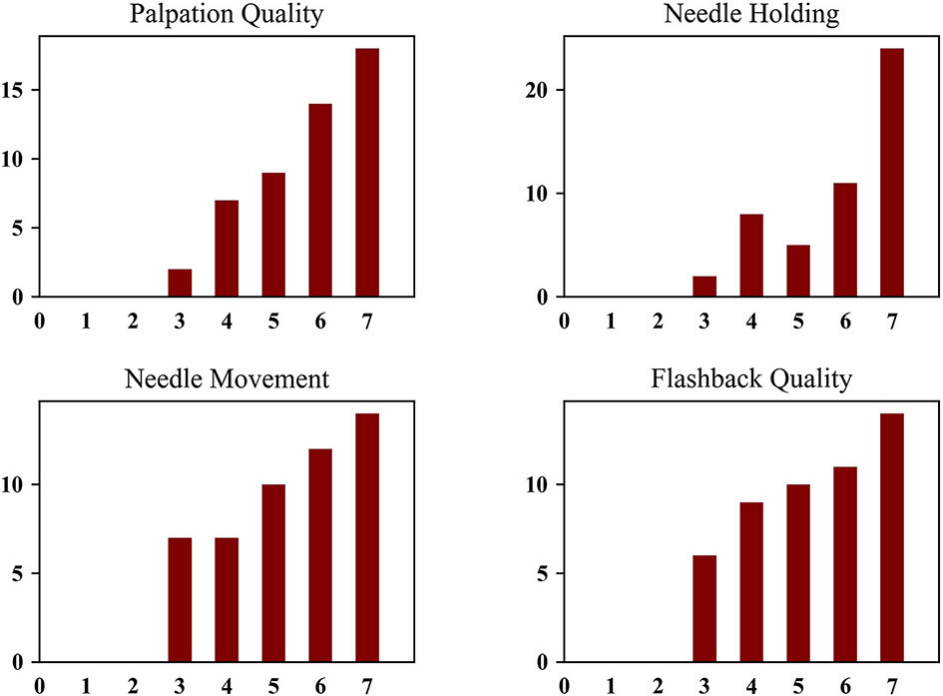
GRS of Individual Categories (Note: inexperienced = 1, low borderline competence = 2, high borderline competence = 3, low average competence = 4, high average competence = 5, low excellent competence = 6, high excellent competence = 7).

**Figure 7:**
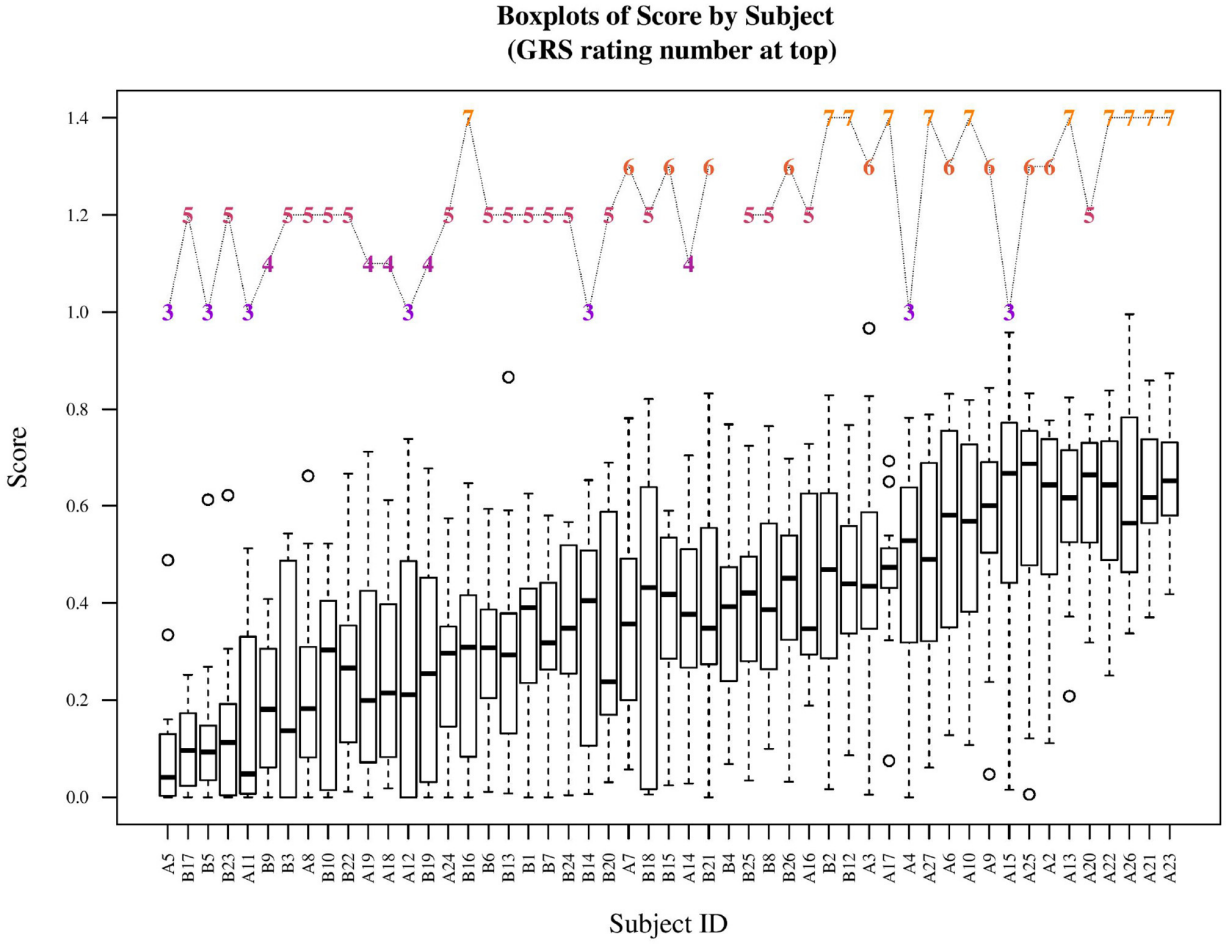
The outcome scores for each subject along with the overall GRS rating. (Note: subjects are sorted by their mean outcome scores.) One participant (B4) did not have a GRS scoring sheet since no preceptor was available at that time.

Figure 6 demonstrates the distribution of GRS scores in each category. In general, there is some correlation between the objective and subjective metrics (*r* estimate: 0.369, *p* < 0.001; 95% confidence interval: [0.308, 0.428]). In Figure 7, the individual participant distributions of their *ocScore* are depicted against their overall GRS scores. (Note that they are ordered by mean *ocScore*, in ascending order.) It can be noted that the two measures of skill generally correlate with each other, with higher mean *ocScore* values correlating with higher overall GRS scores. This suggests that the two metrics validate each other. However, some caveats also must be mentioned. It is readily apparent that there is a relatively high variability of performance (as measured by *ocScore*) among the 16 trials. That is, cannulation performance varied among the 4 different fistulas used as well as on the same fistula. Since GRS measured overall cannulation performance, it was unable to discern differences in performance as a function of fistula properties or number of attempts. These results also bring to light that GRS-based assessment is affected by the limitations of human observation. In some cases, participants with high mean *ocScores* were rated lower by preceptors and vice versa. Participant B16 illustrates this well: though this subject had consistently poor outcome scores (most of the 16 trial scores were below *ocScore* = 0.4), this participant received the highest rating from the preceptor. Similar findings can be observed in participants B23 and A15. Furthermore, subjective biases and variability between preceptors is also evidenced in our results. For instance, though participants A20 and A13 had similar *ocScore* means, since two different preceptors rated their performance via GRS, they received overall ratings of 5 and 7, respectively. A similar effect due to subjective bias arising from two different preceptors can be seen in participants B21 and A14. Another clear difference between traditional manual rating sheet (or GRS) and the objective outcome metric (*ocScore*), is that the *ocScore* can differentiate the quality of performances among the same test subject consistently, while GRS provides a “gross” estimate of the skill level. Consistency during cannulation is a highly desirable characteristic from a patient perspective. Summative GRS assessment that aggregates skill based on a number of cannulation trials is limited in this regard. On the other hand, per-trial GRS assessment (using the rating sheet for each cannulation trial) may be infeasible logistically and economically.

### 3.3. Relationship Between Metrics and Experience

**Figure 8:**
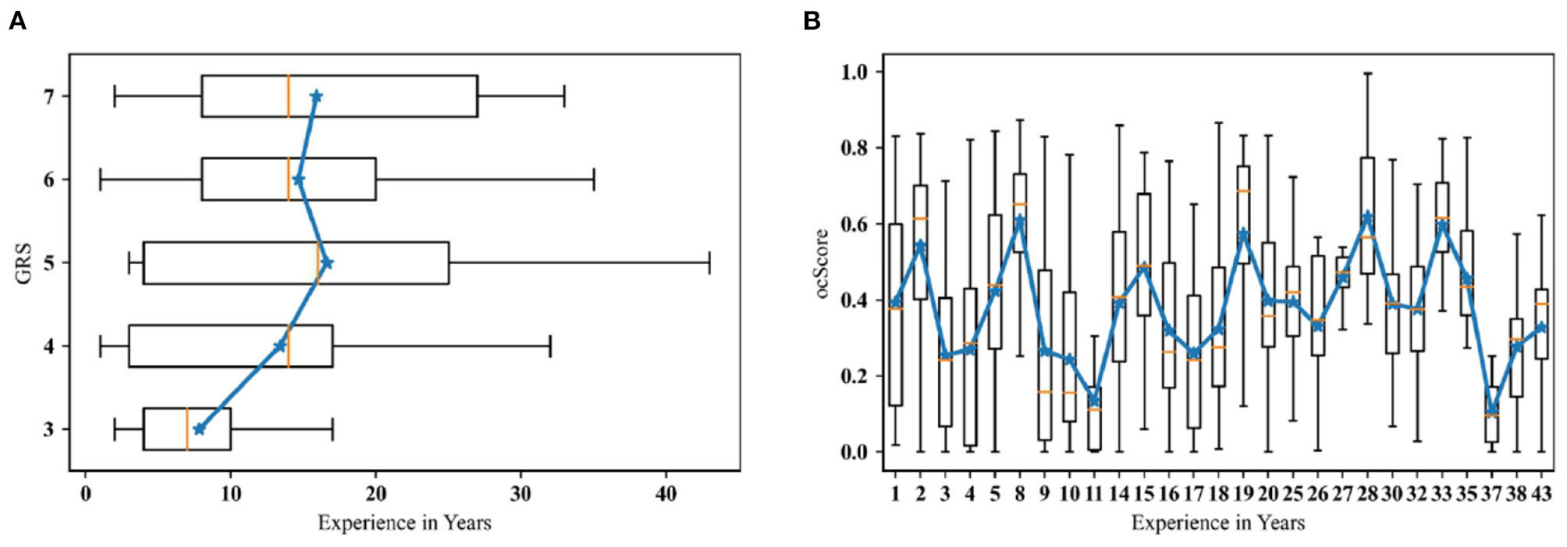
**(A)** Years of experience grouped by GRS; **(B)** The outcome score grouped by experience. (Note: Experience is rounded to full years; solid blue lines connect the means of each group).

It is often implicitly assumed that the expertise level of clinicians is proportional to their years of experience in clinical practice. In this study, we examined the correlation between participants years of experience (acquired through the demographic questionnaire prior to the test) and their assessment metrics. In Figure 8A, mean GRS scores at each level are related to years of clinical experience. The Pearson correlation coefficient was 0.17 at *p* < 0.001, with 95% confidence interval at [0.104, 0.240]. From this, only a marginal correlation between experience and GRS scores is evident. Similarly, Figure 8B presents boxplots of outcome score (*ocScore*) values ordered by years of experience (rounded to the closest integer). The means of each boxplot is connected to provide a quick indicator of a correlation, if any. The Pearson correlation coefficient was 0.010 with *p* = 0.778 (95% confidence interval: [–0.060, 0.079]), indicating no correlation between the objective outcome metric and years of experience. As can be observed, some subjects with less than 10 years of experience have consistently scored above *ocScore* = 0.5, while some subjects with more than 30 years of experience have consistently scored below *ocScore* = 0.25. These results indicate that the common assumption that clinical experience necessarily results in improved skill may be unfounded. This is especially noteworthy since both the subjective and objective metrics demonstrated marginal to no correlation with clinical years of experience.

## 4. Discussion

To reduce the subjectivity of conventional skill assessment that relies on the presence and judgement of an expert educator, we introduce alternative outcome metrics that are simulator-based and objective. Furthermore, the composite outcome metric was constructed based on recently released KDOQI guidelines for skilled hemodialysis cannulation. From our study, the objective outcome-based skill assessment metric has some definite advantages over the subjective assessment method using a Likert-style rating sheet. We discuss the strengths and limitations of both methods in this section.

The GRS method of skill assessment utilizes the expertise of peer-recognized expert preceptors to evaluate the cannulation skill of participants. A key advantage of this method is that it can be used for both simulator-based and in-clinic assessment while the objective outcome metric can be used solely on a simulator. The GRS rating sheet also itemizes cannulation skill into several sub-categories such as palpation, needle motion, etc., which collectively comprise the aspects of cannulation. However, GRS also has some clear limitations. Since GRS is based on human observation, the limitations of human perception are inherent to this type of assessment. Another related aspect is that of human subjectivity and preference, where one preceptor may rate certain or all aspects of cannulation skill higher or lower relative to other preceptors. In light of this, some studies that utilize GRS-like assessment instruments also report inter-rater reliability to address the aspect of human bias and subjectivity. Another related limitation of this method is the resolution at which skill is assessed. While the GRS rating sheet includes several aspects of skill, the weight and meaning of each category will vary depending on the preceptor. Finally, in our study, GRS was used as a gross measure of skill; that is, each participant received 1 overall set of GRS scores (1 overall and 4 skill subcategory scores) since it was deemed infeasible to assess GRS scores for all 16 trials.

Based on our analysis, it is important to note that GRS and ocScore have moderate correlation for cannulation skill assessment. This was expected since GRS assessments are made by preceptors who are exceptional nurse educators who understand the nuances of cannulation skill. Furthermore, while the KDOQI guidelines explicitly articulate what constitutes skilled cannulation (one needle insertion attempt with no infiltrations and stable blood flashback), nurse educators seem to work on this definition implicitly. As can be noted from Figure 6, when following the trend of GRS from left to right the means of *ocScore* (by participant) generally gradually increase from 3 to 7, correspondingly. Clearly, however, there are exceptions. Nevertheless, based on our study, one can conclude that GRS is generally correlated to *ocScore* and, therefore, useful for skill assessment.

The outcome score metric, on the other hand, demonstrates several advantages over GRS scores. Perhaps the greatest strength of the metric is that it is objective, in contrast with the subjective GRS metrics. Further, specific aspects of cannulation skill (needle insertion attempts, number of infiltrations and stable or unstable flashback) are quantified through these metrics. This kind of specificity allows for novice learners as well as educators to identify what particular skills are lacking in a participant. In addition, skill can also be studied as a function of fistula parameters (diameter, curvature, location of needle insertion) with this level of detail. Another strength of the *ocScore* metric is the ability to measure variability of performance even within a subject at a per-trial (vs. per-subject) resolution. Since GRS may not be feasible or ideal for per-cannulation assessment (using the sheet for every trial), an objective, per-trial metric is better suited for training and assessment. In addition, the *ocScore* metric measures skill with a fuller picture of cannulation performance since needle behavior with respect to the fistula is measured by the sensors while preceptors have more limited information from human observation during cannulation. Given the above strengths, as well as the results reported in this study, we believe *ocScore* is superior to GRS for cannulation skill assessment.

An example of the inherent limitation of human observation-based GRS ratings is seen in a few scenarios in our study. As mentioned earlier, the majority of *ocScores* of participant B16 lie below 0.4 (from Figure 6); however, the preceptor rates this person's overall skill with the highest skill rating. This type of incongruence may be because of several reasons including inattention, lack of holistic information about performance, stylistic preferences during cannulation, and other social biases. On the other hand, *ocScore* is formulated to only measure key aspects of cannulation skill. If more than one insertion attempt was detected during cannulation and/or infiltrations occurred combined with low flash efficiency, the resultant *ocScore* was low. This seems to be the case with subject B16, whose scores mostly fell below 0.4. The cases of B23 and A15 are also akin to the above example, demonstrating a difference in how GRS and *ocScore* can yield different assessments of skill. Due to these reasons, we conclude that the objective metric is more desirable and meaningful for skill assessment.

Finally, we wish to address the notion of implicitly equating clinical experience with skill expertise based on our results. While this is commonly used when examining skill, particularly in surgery (15–18), our results call this assumption into question. That is, clinical experience may not necessarily mean a more skilled clinician. In the field of hemodialysis cannulation, Wilson and colleagues have brought to light that many cannulators remain as “perpetual novices” because of the lack of targeted and systematic training opportunities (7). This is particularity important due to the high turnover rate among PCTs in the US as well as the lack of standardization in their training. From our analysis between both the objective and subjective outcome metrics correlation with years of clinical experience, we found a relatively low correlation coefficient between GRS and experience; there was virtually no correlation between *ocScore* and experience. This observation can also be supported by anecdotal evidence from patients, who often voice preference for a specific technician or nurse in a clinic since only he/she knows how to cannulate their fistula well. Consequently, the patient may be filled with anxiety regarding cannulation if that person is not working on a shift. Overall, this novel concept of outcome-based score metric can help reveal the ground truth of cannulation skill. First, the per-trial based metric is able to isolate the performance compared to per-subject based evaluation. Second, this metric is produced by objective outcomes related to KDOQI guidelines. It largely reduces the negative influence of subjective skill assessment. Third, the outcome metric can potentially be useful in skill training by identifying errors. In the climate of value-based medical care, this type of objective skill assessment is not only more cost effective because of the reduced clinical costs due to higher quality care, but also could enable remote learning and assessment. Finally, widespread implementation of this metric has the potential to facilitate standardization of training in the field of hemodialysis cannulation.

There are a few limitations that are worth noting in this study. Results in this report are limited to only the palpation and needle insertion aspects of the hemodialysis cannulation procedure. Of course, other aspects like operating the dialyzer have not been studied. In addition, our simulator features artificial materials for skin, fistulas, tissue, etc., and thus have limitations in realism and functionality. We report data from a set of clinical professionals who, in general, have considerable clinical experience. Last but not least we have not as yet validated our objective and subjective (GRS) scores on the simulator against a real-world assessment of cannulation on a real patient, together with the patients assessment on the cannulation.

To conclude, the results from this study support our initial proposition that the outcome-based score metric demonstrates a higher resolution and less subjectivity compared to manual rating and years of experience.

Most importantly, the ability to improve the quality of cannulation in our hemodialysis units using simulation techniques will make our care more patient-centered since cannulation is important to patients. Also, the ability to cannulate dialysis access grafts and fistulae earlier and without complications will likely reduce the duration of tunneled dialysis catheter contact time, the need for additional endovascular and surgical interventions, and also the cost of care–a win-win situation for all stakeholders involved in ESKD clinical care.

## Data Availability Statement

The datasets presented in this article are not readily available because of restrictions on data access placed by the relevant IRB. Requests to access the datasets should be directed to joseph@clemson.edu.

## Ethics Statement

The studies involving human participants were reviewed and approved by Prisma Health Institutional Review Board. Written informed consent by participants was not required for this study in accordance with the Institutional Review Board.

## Author Contributions

ZL contributed to the system design, data collection, analysis, and drafting and revising of the manuscript. JB contributed to statistical analysis and writing/revising of the manuscript. LP contributed to system design, data analysis, and revising of the manuscript. PR-C, JG, and DB-M contributed to conceptualization, interpretation of results, and writing/revising the manuscript. RS contributed to all aspects of the project including obtaining funding, conceptualization, interpretation of results, and writing/revising the manuscript. All authors contributed to the article and approved the submitted version.

## Funding

This work was supported by the National Institutes of Health/NIDDK grant number K01 DK111767.

## Conflict of Interest

RS reports grant from NIH/NIDDK, during the conduct of the study. He is also Founder of Radiant Ventures, LLC. PR-C is the consultant/Advisory Board member for WL Gore, BD-Bard, Medtronic, Cormedix, Humacyte, Akebia, Bayer, Vifor Pharma and Reata; Founder and CSO of Inovasc LLC. DB-M is an employee of Transonic Systems Inc. The remaining authors declare that the research was conducted in the absence of any commercial or financial relationships that could be construed as a potential conflict of interest.

## Publisher's Note

All claims expressed in this article are solely those of the authors and do not necessarily represent those of their affiliated organizations, or those of the publisher, the editors and the reviewers. Any product that may be evaluated in this article, or claim that may be made by its manufacturer, is not guaranteed or endorsed by the publisher.
